# C1q Deficiency and Neuropsychiatric Systemic Lupus Erythematosus

**DOI:** 10.3389/fimmu.2016.00647

**Published:** 2016-12-27

**Authors:** Rosanne A. van Schaarenburg, César Magro-Checa, Jaap A. Bakker, Y. K. Onno Teng, Ingeborg M. Bajema, Tom W. Huizinga, Gerda M. Steup-Beekman, Leendert A. Trouw

**Affiliations:** ^1^Department of Rheumatology, Leiden University Medical Center, Leiden, Netherlands; ^2^Department of Clinical Chemistry and Laboratory Medicine, Leiden University Medical Center, Leiden, Netherlands; ^3^Department of Nephrology, Leiden University Medical Center, Leiden, Netherlands; ^4^Department of Pathology, Leiden University Medical Center, Leiden, Netherlands

**Keywords:** complement, C1q deficiency, low molecular weight C1q, neuropsychiatric systemic lupus erythematosus, mutation

## Abstract

C1q deficiency is a rare immunodeficiency, which is strongly associated with the development of systemic lupus erythematosus (SLE). A mutation in one of the C1q genes can either lead to complete deficiency or to low C1q levels with C1q polypeptide in the form of low-molecular weight (LMW) C1q. Patients with C1q deficiency mainly present with cutaneous and renal involvement. Although less frequent, neuropsychiatric (NP) involvement has also been reported in 20% of the C1q-deficient patients. This involvement appears to be absent in other deficiencies of early components of the complement classical pathway (CP) (C1r/C1s, C2, or C4 deficiencies). We describe a new case with C1q deficiency with a homozygous G34R mutation in C1qC-producing LMW-C1q presenting with a severe SLE flare with NP involvement. The serum of this patient contained very low levels of a LMW variant of C1q polypeptides. Cell lysates contained the three chains of C1q, but no intact C1q was detected, consistent with the hypothesis of the existence of a LMW-C1q. Furthermore, we provide a literature overview of NP-SLE in C1q deficiency and hypothesize about the potential role of C1q in the pathogenesis of NP involvement in these patients. The onset of NP-SLE in C1q-deficient individuals is more severe when compared with complement competent NP-SLE patients. An important number of cases present with seizures and the most frequent findings in neuroimaging are changes in basal ganglia and cerebral vasculitis. A defective CP, because of non-functional C1q, does not protect against NP involvement in SLE. The absence of C1q and, subsequently, some of its biological functions may be associated with more severe NP-SLE.

## Introduction

C1q deficiency is a rare autosomal recessive-inherited defect of the complement system caused by mutations occurring in one of the three C1q genes (*C1qA*; *C1qB*; and *C1qC*) (1). Up to date, three different categories of mutations according to C1q level have been described. Apart from non-sense mutations and missense mutations leading to absence of C1q in serum, a missense mutation with detectable C1q levels has been described (2). In the last case, some authors have demonstrated a low gradient density of C1q compared with healthy controls and is, therefore, called low-molecular weight (LMW) C1q (3, 4). Until now, a total of 77 C1q deficiency patients in 49 families have been described (5–7). An important variability in clinical presentation and outcome of these patients has been observed, ranging from asymptomatic patients to life-threatening encapsulated bacterial infections (7–9). C1q deficiency is also strongly related to systemic lupus erythematosus (SLE), being so far the most penetrant genetic factor predisposing to this disease. From all patients described, a total of 85% presented SLE-like symptoms while around 50% have been addressed as SLE according to the American College of Rheumatology diagnostic criteria (1, 3, 4, 7, 8). Cutaneous involvement, oral ulcers, and renal involvement are the most consistent manifestations. Although nervous system involvement is less frequent, with only 15 patients described, it can lead to severe neuropsychiatric (NP) symptoms.

Several reports, based on mouse models and/or *in vitro* experiments, describe that C1q plays a role in the brain during different developmental stages. C1q can be neuroprotective in the context of neurotoxicity induced by beta-amyloid (10, 11), but it is also reported to be involved in damage in the context of Alzheimer’s disease (12). It remains to be established to what extent C1q is involved in cognitive (dys)function in humans and how and in which stages of development C1q is protective or damaging to brain tissue.

In this report, we describe a new C1q-deficient patient with a G34R mutation in the C1qC chain leading to severe NP-SLE and review 15 SLE cases with C1q deficiency and NP involvement in the literature. Furthermore, we analyze the biochemical structure of LMW-C1q in serum and in cell lysates.

## Patient and Methods

### Clinical Presentation of the C1q-Deficient Patient

A 24-year-old Dutch man was admitted to our hospital with a 2-day history of progressive weakness and sensory loss of the left arm, visual field loss on the left side, and subjective cognitive complaints with regard to concentration and memory. He had been diagnosed with a SLE-like illness associated with C1q deficiency at the age of 10 months when he presented a butterfly rash and antinuclear antibodies (ANAs) positivity. The C1q deficiency was caused by a homozygous g.5499G>A mutation at the C1qC gene, resulting in a G34R change in the C1q protein. Consanguinity was not reported.

At the age of 3 years, he developed polyarthritis, which was successfully treated with naproxen. At the age of 7 years, he was admitted due to a relapsing polyarthritis and subacute cutaneous lupus, fever, aphthous ulcers, sunlight hypersensitivity, malaise, and positive antibodies including ANAs, anti-Ro, anti-RNP70, and Sm. SLE was diagnosed and hydroxychloroquine 200 mg was started. Examination of the past medical history also included frequent upper airway and ear infections during the first 3 years of his life, pertussis infection at the age of 4 years, relapsing impetigo with a *Staphylococcus aureus* septicemia at the age of 19 years and relapsing virus *Varicella zoster* infection after the age of 20 years.

**Figure 1:**
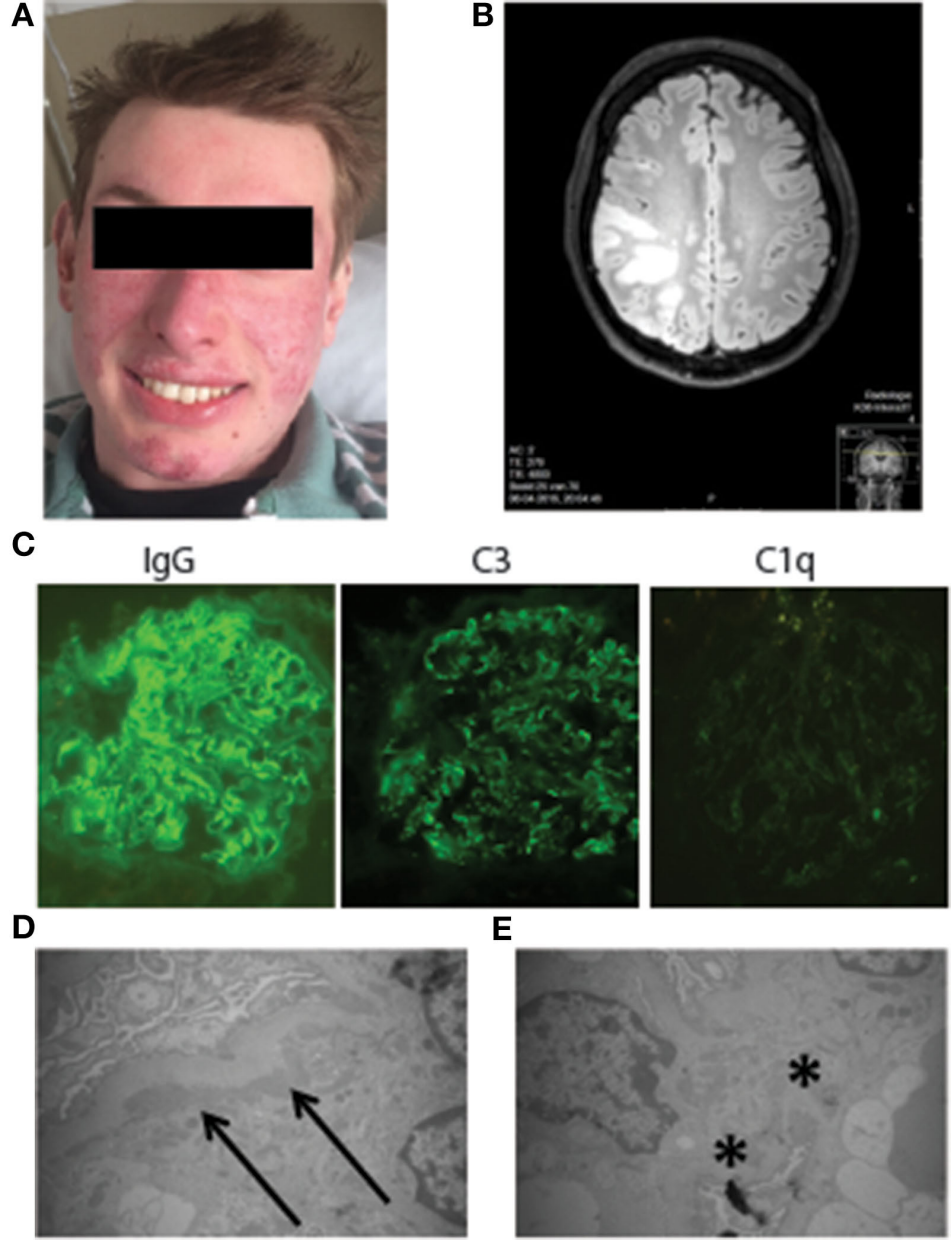
**Clinical presentation of the C1q-deficient patient**. **(A)** Malar rash and discoid lupus leading to mild scarring and atrophy. **(B)** The 3-T magnetic resonance imaging brain (FLAIR image): multifocal diffuse gray matter hyperintensities located in the frontotemporal right lobe and high-intensity area in multiple regions of the right frontal and parietal lobes. **(C)** Immunofluorescence staining of IgG deposition, C3 deposition, and C1q deposition on the kidney. **(D)** Electron micrograph of the subendothelial deposition (arrows) of electron dense material. **(E)** Electron micrograph of mesangial deposition (stars) of electron dense material.

On the current admission, the patient’s body temperature was 37.7°C and blood pressure was 100/60 mmHg. Physical examination was remarkable with a butterfly rash (Figure 1A), severe sensory loss of the left arm, hyperesthesia of the left hand, and homonymous hemianopsia of the left side. Laboratory tests revealed increased ESR (63 mm/h; normal <15) and CRP (13.7 mg/L; normal <5), a normal hemoglobin, and complete blood count. Except for a reduced serum albumin level (31 g/L; normal 34–48), electrolytes, serum cholesterol, renal, and liver testing were normal. Analysis of the urine was normal without casts or dysmorphic red cells. Protein excretion was 9.87 g/24 h. The antibody profile was positive for ANAs, anti-Ro (>240 U/mL, normal <7), anti-RNP70 (79 U/mL, normal <5), and anti-Sm antibodies (>120 U/mL, normal <5). Anti-double-stranded DNA, anticardiolipin antibodies, Beta-2-GP1 antibodies, lupus anticoagulant, anti-phospholipase-A2-receptor (PLA2R), and anti-C1q autoantibodies were negative. At this time, analysis of complement showed a classical pathway (CP) activity of 0% (normal >74%), a low alternative pathway (AP) activity (22%, normal >39%), a low level of C1q (21 mg/L, normal 102–171 mg/L), whereas C3 (1.4 g/L, normal 0.9–2.0 g/L), and C4 (396 mg/L, normal 95–415 mg/L) were in the normal range. Blood and urine cultures were negative. Findings from the renal biopsy were compatible with a class V lupus nephritis, with a “nearly full house” immunostaining showing a strong granular staining for IgG and a moderate granular staining for C3, both along the glomerular basement membrane; a slight granular staining for IgA and IgM, and kappa and lambda light chains, sometimes also in mesangial areas, but no staining for C1q (Figure 1C). Electron microscopy revealed subendothelial, subepithelial, and mesangial deposits (Figures 1D,E). A low minimental state examination for the age and education of the patient (24, range 0–30) was found. A brain computed tomography (CT) scan demonstrated a hyperdensity at the right frontal and parietal lobes and a contrast enhanced CT showed a bilateral filling defect in the transverse sigmoid sinus. A magnetic resonance imaging (MRI) showed multifocal diffuse gray matter hyperintensities located in the frontotemporal right lobe and high-intensity area on T2 in multiple regions of the right frontal and parietal lobes with high-intensities on the diffusion weighted imaging study (Figure 1B). A CT-angiography showed no signs of cerebral vasculitis. A diagnosis of lupus nephritis type V and NP-SLE with both inflammatory and ischemic phenotype were established. The patient was treated with daily clopidrogrel 75 mg and intravenous methylprednisolone 1 g for 3 days plus oral prednisone 1 mg/kg/day in a tapering dose, and monthly intravenous cyclophosphamide 1 g/m^2^ for 6 months. Proteinuria improved dramatically in the first week and homonymous hemianopsia and cognitive dysfunction resolved after 2 weeks. After 3 months, the patient still presented a mild sensory loss of the left arm. Both the patient and his parents provided informed consent for the studies.

### Samples

Serum and PBMCs, isolated by Ficoll–Paque density gradient centrifugation were collected from the patient and an age-matched control. During the admission, a kidney biopsy was performed.

### Microscopy

Slides for light microscopy evaluation were stained by hematoxylin and eosin, PAS, and silver staining. Immunofluorescent stainings on cryostat sections were performed for IgA, IgG, IgM, C3, C1q, and kappa and lambda light chains. Part of the renal specimen was used for electron microscopy. Pictures were taken with a JEM-1011 electron microscope (JEOL USA, Inc.) at various magnifications.

### Gel Filtration

Gel filtration experiments were carried out using the Äktaprime plus system (GE Healthcare, 11001313). Five hundred microliters of filtered serum sample, either the healthy control serum or serum from the C1q-deficient patient, was run through a Hiload Superdex Prep grade 200 16/600 column (GE Healthcare), using PBS as the running buffer. Fractions of 1 mL were collected starting after half an hour for the duration of approximately 50 fractions. The protein levels in the fractions were analyzed using a Pierce^TM^ BCA Protein Assay Kit (ThermoFisher Scientific).

### C1q ELISA

The levels of C1q in serum and supernatants were measured using an in-house developed ELISA. Maxisorp plates (Nunc) were coated with mouse anti-human C1q (Department of Nephrology, LUMC) in coating buffer (0.1 M NA2CO3, 0.1 M NaHCO3, pH 9.6) overnight at 4°C. Plates were washed in PBS/0.05% Tween (PBS-T, Sigma). Then the wells were blocked with PBS/1% BSA for 1 h at room temperature. After washing, the patient serum and control serum were added to the wells in a twofold dilution series starting from 1:100 diluted in PBS/1% BSA/0.05% Tween (Sigma). After incubation for 1 h at 37°C, the plates were incubated with rabbit anti-human C1q (DAKO) for 1 h at 37°C and as detection antibody goat anti-rabbit HRP (DAKO) was used. Finally, the substrate was added using ABTS (sigma). The C1q levels were measured at an absorbance level of 415 nm.

### Western Blot

Using western blot, the composition of C1q was examined by detection of the three chains of the C1q protein. Due to the low amount of C1q present in the serum of the patient, we applied 10 times more serum of the patient than the healthy donor. Cell lysates and supernatants of stimulated and unstimulated PBMCs of the healthy control and the patient were used in the same amount in reduced and non-reduced SDS conditions. The western blot was performed using previously described methods (9).

### Reconstitution Complement Activity Assay

To exclude the possibility that next to C1q deficient, the patients sample would also be deficient for C1r or C1s, we performed assays to measure activation of the CP of the patient serum by reconstitution of purified C1q. Plates coated with human IgG were incubated with 1% serum of the patient (diluted in GVB++; 0.1% gelatin, 5 mM Veronal, 145 mM NaCl, 0.025% NaN3, 0.15 mM CaCl, 0.5 mM MgCl, pH 7.3) with or without addition of purified C1q (Quidel) in different concentrations. As a read-out, C4 deposition was measured.

### Sequencing

Genomic DNA was extracted from blood collected with tubes supplemented with EDTA. Sequencing of the complete C1q genes (*C1qA, C1qB, and C1qC*), of both introns and exons was performed as before (9). Deep-sequencing was performed using the 454 NGS Roche GS FLX Titanium platform. Data were compared to internal controls and to Human Genome build 19 as well as Human_v37_2 de dbSNP database v132 using the NextGENe software package for Next Generation Sequence Analysis (NGS) from Softgenetics. The effect of the mutation on splicing was *in silico* analyzed using the NetGene2 Server, http://www.cbs.dtu.dk/services/NetGene2/.

## Results

### Detection of LMW-C1q in Serum

**Figure 2:**
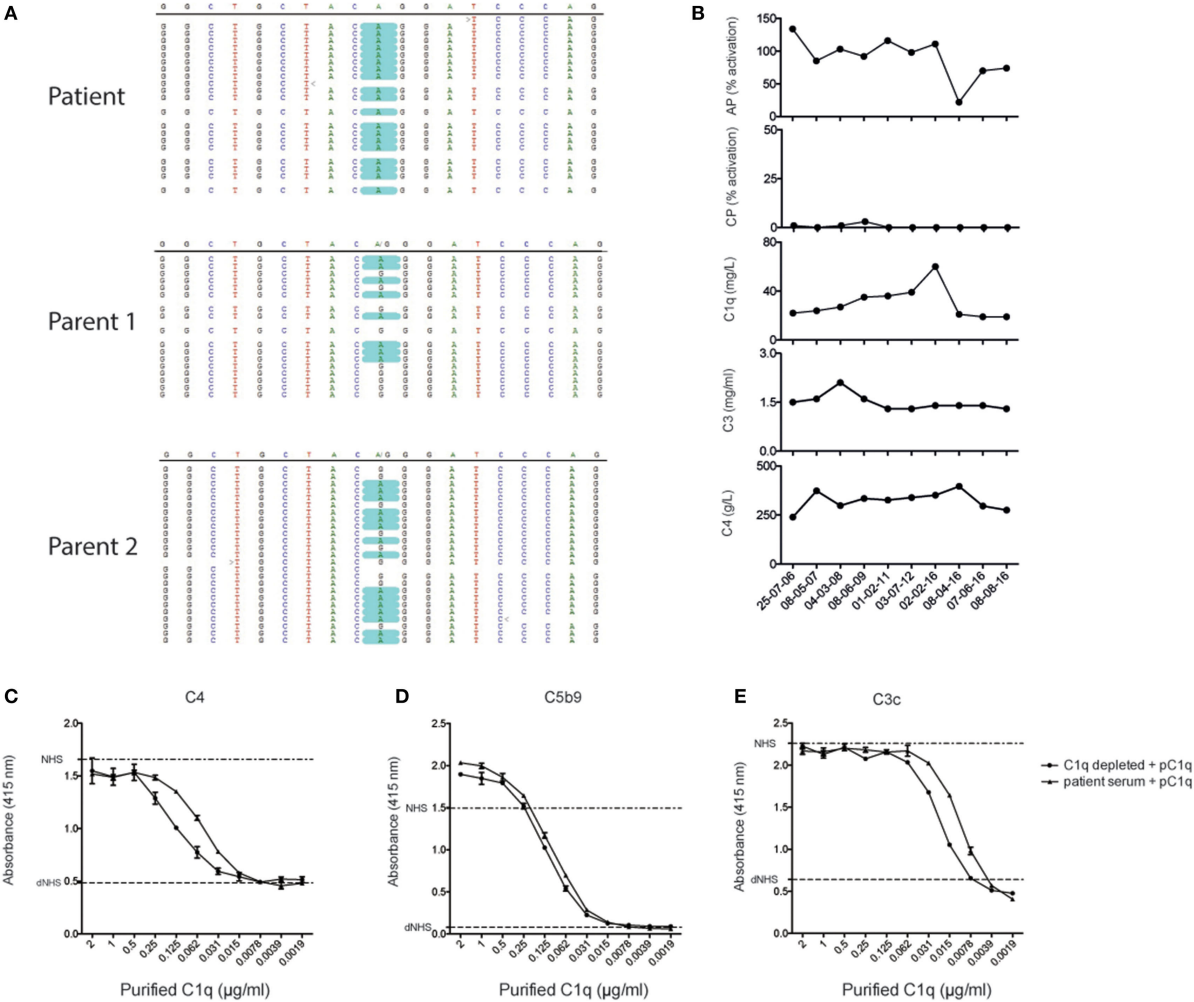
**Genetic analysis of the patient and complement activation assays**. **(A)** Data obtained from deep sequencing show a G34R mutation in the C1qC chain. **(B)** Measurement of the alternative pathway (Wieslab), classical pathway (CP) (Wieslab), C1q, C3, and C4 with nephelometer measurement in the diagnostic laboratory. **(C)** Reconstitution of the CP by adding different concentrations of purified C1q to the patient serum. As a positive control, normal human serum (NHS) was used, and as a negative control, heat-inactivated NHS (ΔNHS) was used. C4 deposition was used as detection antibody. **(D)** C5b9 deposition after adding purified C1q to the patient serum and C1q depleted serum. **(E)** C3c deposition.

With deep sequencing, we identified a homozygous g.5499G>A mutation in the *C1qC* gene, resulting in a change in the C1qC chain where glycine was changed into an arginine at position 34 (G34R), while both parents show a heterozygous state of the mutation (Figure 2A). The routine diagnostics laboratory reported the patient to be completely lacking CP activity (Figure 2B). This is compatible with a C1q deficiency, but to exclude that next to C1q also other factors would be deficient in the patient, we performed a reconstitution assay where we add purified C1q to the serum of the patient and analyze C4 deposition. To compare the activity, we performed the same analysis with C1q-depleted serum. After adding purified C1q, we were able to detect C4 deposition at a similar range as C1q-depleted serum reconstituted with pC1q (Figure 2C). This indicated that the patient was able to produce C1r and C1s, C2 and C4, and together with purified C1q was able to activate the CP. Furthermore, we were also able to measure C5b9 and C3c deposition. This implied that there were no other complement deficiencies downstream in the complement system (Figures 2D,E).

**Figure 3:**
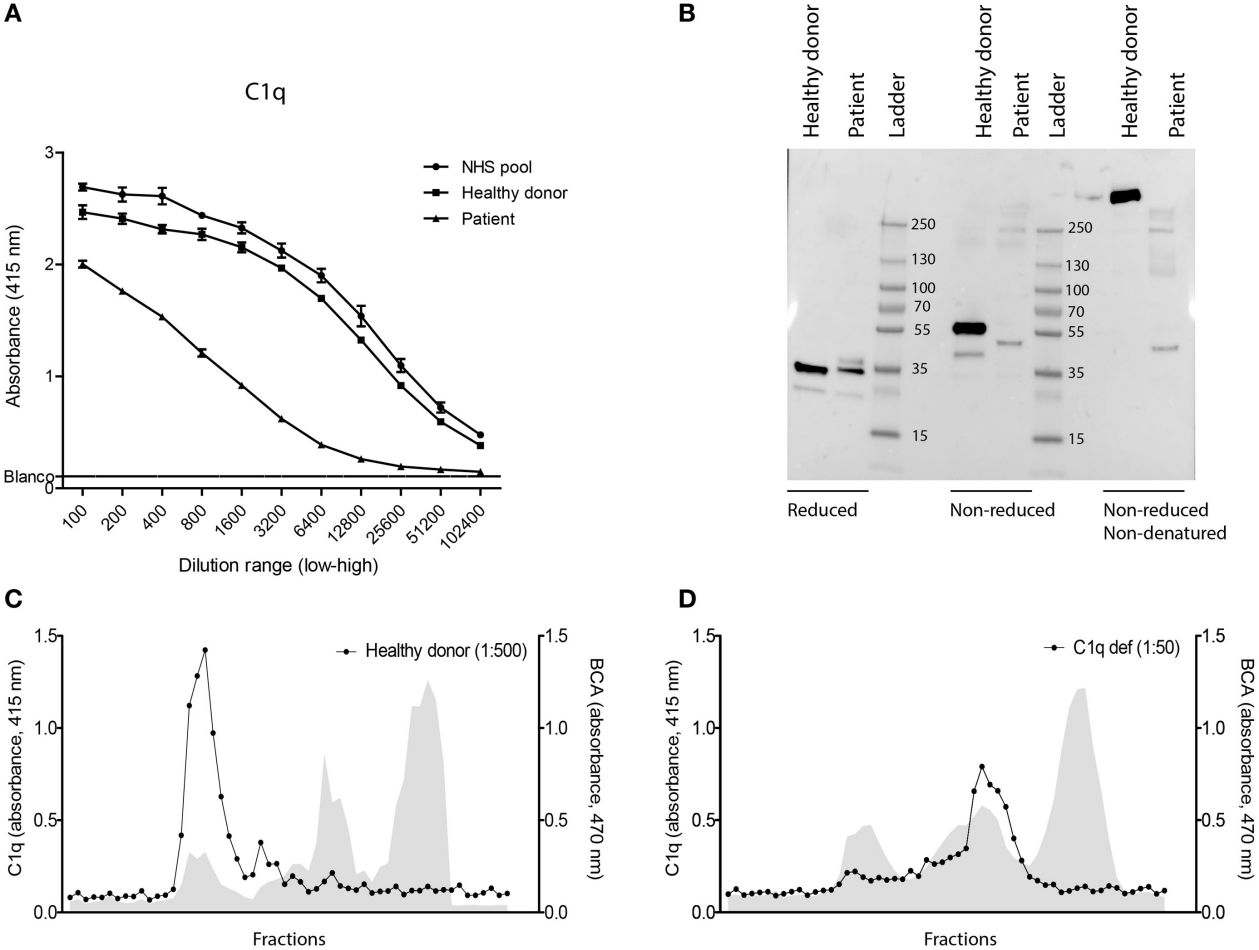
**Detection of low-molecular weight-C1q in serum**. **(A)** C1q ELISA by using a dilution range of the serum of the C1q-deficient patient (▲), age-matched control (■), and normal human serum (●) as extra control. **(B)** Western blot analysis of the serum in reduced, non-reduced, and non-reduced/non-denatured conditions. As positive control, an age-matched control is used. Patient serum was diluted 50× and the healthy control 500×. **(C)** Protein analysis using a BCA protocol and C1q ELISA of different fractions after gel filtration of the serum of a healthy donor. **(D)** Protein and C1q analysis of the patient.

Using ELISA, we could detect a decreased amount of C1q in the patient compared to the control samples (Figure 3A). We used western blot to examine the molecular structure of C1q in the patient serum. In reducing conditions, all the three chains of the correct size are detected. However, using non-reducing conditions, the dimers of C1q (2 × A–B and 1 × C–C) show an abnormal pattern. Using non-reducing/non-denaturing conditions, we were able to detect high-molecular weight C1q in the healthy control, but not in the patient, suggesting that the C1q of the patient is of a LMW species (Figure 3B). With the usage of gel filtration the serum samples of the healthy donor and the patient were fractionated on size and with a BCA, the amount of protein was analyzed. While the protein profiles of both gel filtrations are similar, the location of C1q in the elution profiles is clearly different (Figures 3C,D). Please note that since the serum of the patient was very low in C1q concentration, we had to use different dilutions for the patient and the control in the ELISA to detect the presence of C1q in the fractions. These size-exclusion chromatography data confirm the LMW nature of C1q in the serum of the patient.

### Composition of C1q in PBMC of the C1q-Deficient Patient

**Figure 4:**
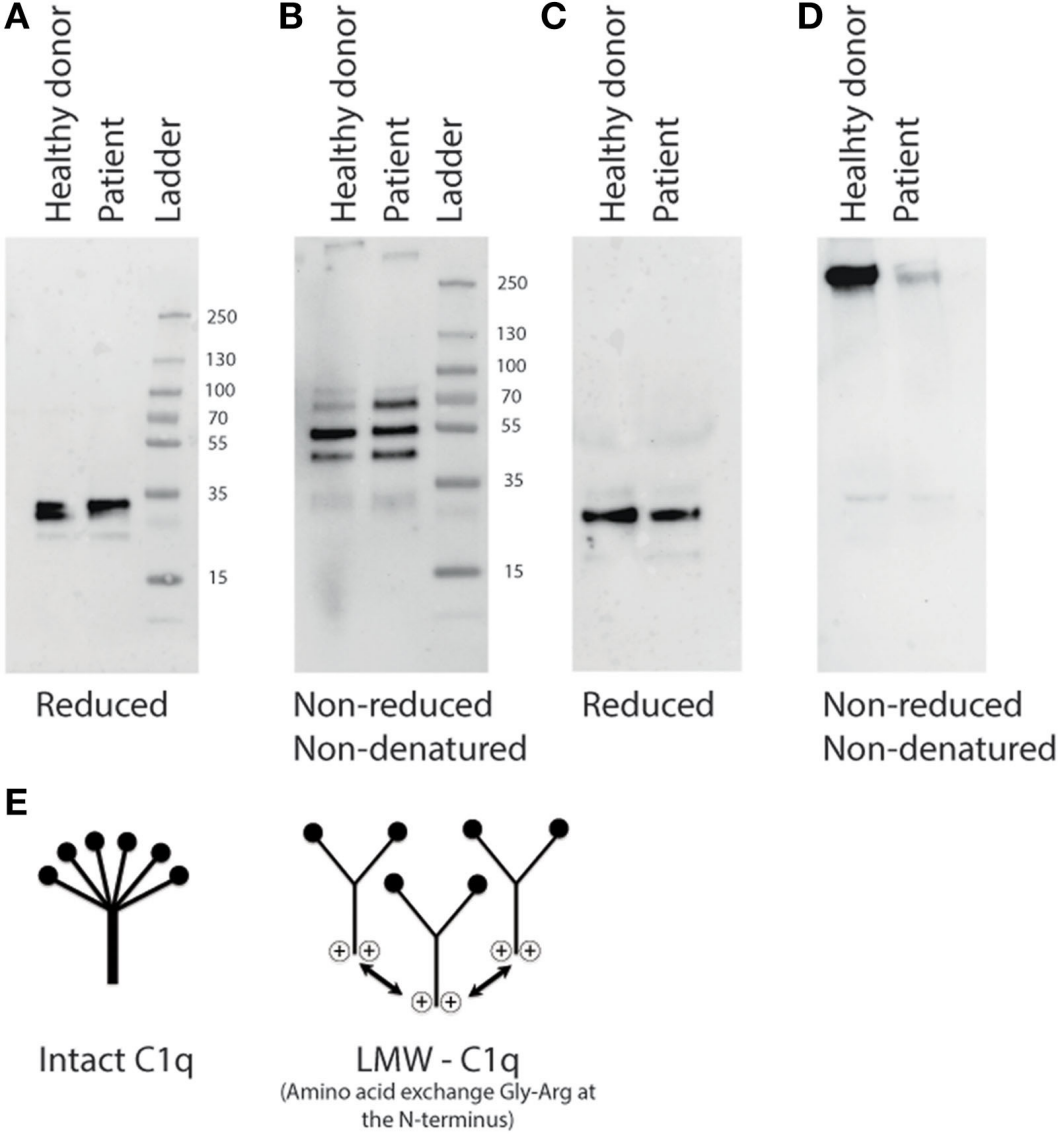
**Analysis of stimulated cells from the C1q-deficient patient on the presence of C1q, (A) Western blot analysis of cell lysates from stimulated PBMCs in reducing conditions, (B) non-reducing and non-denaturing conditions**. **(C)** Western blot analysis of the supernatant of the PBMCs from the patient and the healthy donor (control) after 72 h of culturing in reducing conditions. **(D)** In non-reducing and non-denatured conditions, the cell lysates and supernatant were added in the same amount. **(E)** A schematic representation of intact C1q and low molecular weight (LMW)-C1q. In LMW-C1q, positive charges are introduced in the collagen-like tail due the amino acid exchange Gly-Arg at the N-terminal region [modified from Ref. (2)].

To further examine the production of C1q by the cells of the patient by Western Blot, we stimulated PBMCs of the patient and the control with DXM and IFN-γ to upregulate the C1q production. Compared to the serum, we loaded the same amount of lysate and supernatant to the lanes. In reducing conditions, we see all the three C1q chains in the lysate of the PBMCs (Figure 4A). The dimers of C1q can also be detected in the lysates of the PBMCs from the patient. However, in non-reducing and non-denaturing conditions, the dimers of C1q are detected, while additional bands are seen in the PBMCs of the patients, which may indicate the presence of intracellular LMW-C1q (Figure 4B).

To examine the composition of secreted C1q, the supernatant of the PBMCs was analyzed using western blot. The three chains of C1q were detected in the control supernatant as well as in the patient supernatant in reducing conditions. Surprisingly, the amount of C1q seems comparable between the patient and the control (Figure 4C). In non-reducing, non-denaturing conditions, the high molecular size of C1q (460 kDa) is detected only in a very low concentration compared to the supernatant of the healthy control (Figure 4D).

### C1q Deficiency and NP-SLE

We performed an extensive electronic literature search from 1980 to 2016 using online databases (PubMed, Embase, and Medline). We found 15 C1q-deficient patients with NP-SLE. All these patients presented at least one major central nervous system (CNS) manifestation. Clinical and neuroimaging characteristics of these patients are summarized in Table S1 in Supplementary Material. Among all C1q-deficient patients with NP-SLE described so far in the literature, seizures was the most frequent NP symptom presented (10 patients; 67%) (6, 13–20). Furthermore, five patients (33%) presented with a series of severe non-specific NP symptoms characterized by encephalopathy and difficulties to walk associated with cerebral infarcts and thought to be related with a cerebral vasculitis (5, 13, 19–21). Transverse myelitis (6, 22) and psychosis (14, 22) were also present in two patients (13%). Neuroimaging of the brain showed as more frequent finding affection of basal ganglia (calcification or ischemic lesions) in 40% of the cases (16, 17, 19–21, 23) followed by cerebral vasculitis (27%) (13, 15, 20, 21) and brain atrophy (20%) (6, 17, 24).

## Discussion

The present study investigated an extremely rare case of C1q deficiency due to non-functional LWM-C1q associated with a severe clinical phenotype presenting with membranous lupus nephritis and a mixed inflammatory and ischemic NP-SLE. C1q deficiency is a very strong susceptibility factor for the development of SLE where patients mainly present during childhood with skin or renal involvement and less frequently also with NP involvement (7). Interestingly, although all the deficiencies of early components of the complement CP are known to be a susceptibility factor for the development of SLE-like disease, NP involvement appears to be absent in C1r/C1s, C2, or C4 deficiencies (24, 25). This makes us to speculate about the possible role of C1q in the underlying process leading to NP-SLE.

Neuropsychiatric involvement in SLE-related C1q deficiency presents with severe major CNS manifestations and its prevalence seems to be slightly higher than in complement competent NP-SLE patients (20 vs. <5%) (26). Seizures were the most common manifestation, presented in 60% of NP-SLE patients. In animal models, the production of C1q by neuronal cells was reported to lead to opsonization of synapses in the developing postnatal CNS, which are next eliminated by microglia (27). Several studies in murine models have described that C1q plays a role in the brain during different developmental stages. A neuroprotective role for C1q was reported in the context of beta-amyloid-induced neurotoxicity (10, 11), while on the other hand, C1q is reported to be involved in damage in the context of Alzheimer’s disease (12). The complement system can hence facilitate normal neuronal development and protect against damage or contribute to neurodegenerative disease depending on yet to be identified triggers and timing. Currently, it has not been formally studied whether C1q-deficient patients have cognitive impairments. The neurological status of the current case completely normalized after the successful treatment of the SLE flare with immunosuppression, without any residual cognitive impairment. Moreover, studies using C1q knockout mice have demonstrated how a defective neocortical pruning of excessive excitatory synapses in these animals results in spontaneous and evoked epileptiform activity and increased intracortical excitatory connectivity (28, 29). This may explain the increased prevalence of seizures among these patients. Of note, neuroimaging demonstrated that a total of 40% of patients with C1q deficiency presenting with NP-SLE showed involvement of the basal ganglia and in 27% of these patients findings were compatible with cerebral vasculitis. Neuroimaging changes in basal ganglia have been rarely reported in SLE patients. It has been suggested that these findings may represent vasogenic edema and vascular changes occurring due to a vasculitic process localized in the basal ganglia probably due to immune-mediated underlying pathogenesis or effect of inflammation. Moreover, these MRI findings have been described to be reversible after starting immunosuppressive therapy (30). SLE-associated vasculitis may be associated with the deposition of immune complexes (ICs) in the endothelium. The deposition of these ICs may lead to endothelial cell activation and inflammatory cell infiltration (31). Previous reports have proposed an important role of C1q in the clearance of apoptotic cells and circulating ICs (32, 33). Non-cleared debris due to absence of C1q may lead to helper T cells stimulation and autoantibody production (34, 35). Furthermore, in the last years, C1q has been demonstrated to be of importance in vascular endothelial permeability and integrity. C1q and mannose-binding lectin have been reported in *in vitro* studies to help in the removal of atherogenic lipoproteins, which has been proposed as a link between C1q deficiency and cardiovascular disease in SLE, as seen in our patient (36, 37).

Globally, more than 60 patients are described with a C1q deficiency mostly due to a homozygous mutation. From these patients, six have the g.5499G>A mutation resulting in a G34R amino acid change and C1q deficiency (4, 14, 16, 17, 20, 38). Previous case reports that described the G34R mutation suggested the development of LMW-C1q, which is known as a non-functional C1q. In this study, we demonstrate a C1q-deficient patient with a low level of circulating C1q and an absence of CP activity recorded over a long time period. Using sequencing, we confirmed a homozygous G34R mutation. As suggested in previous studies, we also observed that the C1q present in this patient is LMW-C1q. Using western blot and gel filtration of the patient serum, we detected a different molecular size of C1q in the patient serum at low concentrations. When we analyzed the production of C1q by PBMCs, we could detect all three C1q chains at a same concentration intracellularly, but after analyzing C1q in the supernatant in non-reducing and non-denaturing conditions, almost no fully folded C1q was detected. This confirms that the patient is able to produce all C1q chains but is unable to fold a complete functional C1q molecule (Figure 4E). It is conceivable that the incorrectly folded C1q polypeptide chains have a strongly reduced half-life. Circulating C1q was completely absent after a flare of NPSLE. This may suggest that there is consumption of the little C1q polypeptide that the patient produces. However, in the renal biopsy, no C1q was detected, which could also indicate that it is not consumption of LMW C1q but rather a reduced production at the time of flare. Although temporary expression of LMW-C1q has been reported to occur during SLE flares or even in healthy persons, this production is temporary and involves only part of the total C1q pool (39, 40). In the current patient, the production of LMW-C1q is genetically regulated and permanent and results in a completely defective CP.

In conclusion, NP-SLE is a rare but severe complication in C1q deficiency patients that must be diagnosed and treated promptly. The low level of LMW-C1q observed in the patient did not allow any CP activity, making the patient functionally C1q deficient. The role of C1q or its absence in the pathogenesis of NP-SLE merits further studies.

## Author Contributions

The first two authors contributed equally to this manuscript. Conception and design: RS, CM-C, TH, GS-B, and LT. Acquisition of data: all the authors. Laboratory processes: RS and LT. Drafting the article: RS and CM-C. All the authors were involved in revising the article critically for important intellectual content and they approved the final version to be published.

## Conflict of Interest Statement

The authors declare that the research was conducted in the absence of any commercial or financial relationships that could be construed as a potential conflict of interest.

## Abbreviations

ABTS, 2,2′-azino-bis-3-ethyl benzthiazoline-6-sulfonic acid; DXM, dexamethason; ECL, enhanced chemiluminescence; ELISA, enzyme-linked immunosorbent assay; HRP, horseradish peroxidase; IFN, interferon; kDa, kilo dalton; NGS, next generation sequence; NHS, normal human serum; NP-SLE, neuropsychiatric systemic lupus erythematosus; PBMC, peripheral blood mononuclear cell; SDS/PAGE, sodium dodecyl sulfate polyacrylamide gel electrophoresis; SLE, systemic lupus erythematosus.
