# Supplementary material for: C1q Deficiency and Neuropsychiatric Systemic Lupus Erythematosus
Source: Front Immunol. 2016 Dec 27;7:647. doi: 10.3389/fimmu.2016.00647 (PMC5186770; doi:10.3389/fimmu.2016.00647)
Supplement: Supplementary file 1 [file Table_1.DOCX]

| **Supplementary Table 1. Cases reported with C1q deficiency and neuropsychiatric systemic lupus erythematosus** | | | | | | | | | | | | |
| --- | --- | --- | --- | --- | --- | --- | --- | --- | --- | --- | --- | --- |
| **Age at onset/Sex/Flare** | **Country** | **Clinical features** | **NPSLE manifestation** | **Notes** | **Immunological tests** | **Complement functional tests** | **Neuroimaging** | **Medication** | **Mutation** | **Consequence/ Type C1q deficiency** | Ref. |  |
| 1/M/ND | Yougoslavia | Malar rash, oral ulcers, photosensitivity, arthritis, LN (MPGN), NPSLE (Seizure) | Seizure | Recurrent infections  Died at  13 | ANA +  DNA+  SSA +  Sm+  RNP+ | C1q=0 | ND | Corticoids, frozen plasma, plasmapheresis and IVIG | g.8626C>T | Arg69X / Complete | [17] |  |
| 13/F/2 | Saoudi Arabia | Malar rash, discoid rash, oral ulcers, arthritis, leukopenia, thrombopeina, alopecia | Seizures, mononeuritis multiplex | ND | ANA+  DNA-  ENA- | C1q=0 | ND | Corticoids, CYC | ND | ND | [18] |  |
| 7/F/7 and 20 | Dutch | Malar rash, oral ulcers, LN, fever, alopecia, lymphadenopathy, myositis | Seizure, hemiplegia and lethargy. Probably cerebral vasculitis | Died  at 20 | ANA+  DNA-  RNP+ | C1q<0.1  C1r/C1s=0  C3/C4: N  CH_50_<1%  C1inh= N | Brain scintigraphy: multiple spots with activity mainly right sided, probably due to vasculitis | Corticoids, CYC | ND | ND / Dysfunctional | [13] |  |
| 9/F/9 | Japan | Malar rash, discoid rash, photosensitivity, oral ulcers, proteinuria (no biopsy), arthralgia | Seizure |  | ANA+  DNA –  SSA +  Sm+  RNP + | ND  LMW C1q | Calcification of the basal ganglia and the temporal lobe (CT-scan) | Corticoids | g.5499G>A | Gly34Arg / Dysfunctional | [16] |  |
| 6/F/18,24 and 29 | Germany | Malar rash, oral ulcers, photosensitivity, leukopenia, pleuritis, arthritis, glomerulonephritis (Type V) and Libman-Sacks endocarditis, peritonitis | Seizure and psychosis | Renal and heart failure died at  29 | ANA+  DNA+  Sm+ | C1q=28%  C1r/C1s=N  C2-C4f =N  C3/C4= N  CH_50_=0  AP_50_=N  C1inh =N | ND | Corticoids, plasmapheresis, chlorambucil, CYC, cyclosporin, IVIG | g.5499G>A | Gly34Arg / Dysfunctional | [14] |  |
| 9/F/25 | England | Malar rash, photosensivity, leukopenia, alopecia | Seizure and cognitive dysfunction | Recurrent infections  Died at 28 | ANA +  DNA –  SSA +  Sm +  RNP + | C1q=0  CH_50_<5%  AP_50_= N | Periventricular and basal ganglia calcification, with severe cerebral atrophy | Corticoids, Azathioprine, frozen plasma and plasmapheresis | g.8633delC | Gln71fsX137 / Complete | [17] |  |
| 5/F/ND | Saudi Arabian | Discoid lupus, photosensitivity, lupus nephritis (non-specified), alopecia | CNS involvement with cerebral atrophy, non-specified |  | ANA +  DNA -  SSA +  SSB +  Sm + | ND | ND. Cerebral atrophy | Unknown | ND | ND / dysfunctional | [24] |  |
| 3/F/3 and 10 | Inuit | Malar rash, discoid rash, photosensitivity, oral ulcers | CNS involvement, non-specified | Died at 10 of Pneumocystis carinii pneumonia | ANA +  DNA -  Sm +  RNP +  RF + | C1q< 6%  C1s/C1r= N  C3/C4= N  CH_50_ =1%  AP_50_=N  C1 inh= N  MBL= N | ND | Corticoids, methotrexate, | g.13166G>A | Gly244Arg / Complete | [23] |  |
| 3/M/10 | Pakistan | Malar rash, oral ulcers, fever | Cerebral vasculitis (Encephalopathy with global dysphasia, quadra and bulbar paresis, generalized hypertonia  and resting tremor) | Bacterial meningitis at 3 | ANA +  DNA -  SSA +  Sm + | C1q=0  C2/C3/C4=N CH_50_=0  AP_50_=N | Bilateral infarction of his basal  ganglia suggestive of a small vessel vasculitis | Corticoids, CYC, IVIG | ND | ND | [21] |  |
| 7/F/7 | Arabian | Discoid rash, arthritis | Seizure, ACS, multiple ischemic lesions | Hyper IgM Syndrome Recurrent infections | ANA +  DNA +  SSA+  SSB+ | C1q=5%  CH_50_=0 | Multiple ischemic lesions involving both white matter and grey matter of hemisphere with left-sided predominance and also basal ganglia | Corticoids, CYC | g.5499G>A | Gly34Arg / Dysfunctional | [20] |  |
| 6/F/6 and 15 | Pakistan | Malar rash, oral ulcers, alopecia, Raynaud, fever and arthralgia | Seizure, cerebral vasculitis | Recurrent infections | ANA+  DNA-  Sm+ | C1q=0 (ELISA)  C3/C4= N  CH_50_=0  AP_50_=N | Left frontal lobe infarct secondary to cerebral vasculitis | Corticoids, Azathioprine, frozen plasma | g.5564delG | Gly55fsX83 / Complete | [15] |  |
| 32/M/ND | Arabic | Thrombopenia, lymphopenia, AIHA, polymyositis | Seizure, transverse myelitis | Recurrent infections  Died of bacterial septic shock and multi-organ failure | ANA+  RNP+  Ribosomal P +  ACA + | C1q= Normal  C2-C9=N  CH_50_=0 | MRI brain and spine: brain atrophy, thoracic spinal cord atrophy | Corticoids, CYC | Codon 48 *Bchain* | Gly63Ser / dysfunctional | [6] |  |
| 1/M/ND | Brasil | Photosensitivity, pericarditis | Psychosis and transverse myelitis | Cutaneous pyogenic infections and septic shock | ANA +  Sm +  RNP + | C1q=0  AntiC1q=0 | ND | ND | ND | ND | [22] |  |
| 1/F/2 and 4 | Maltese | Malar rash, oral ulcers, fever, Raynaud, vasculitic lesions fingers | Seizure, cerebral vasculopathy/vasculitis with several strokes, encephalopathy associated with spasticity | Salmonella infection | ANA +  DNA +  Ribosomal P + | C1q=0  CH_50_=0 | Bilateral frontal infarcts and basal ganglia calciﬁcation. Acquired moya-moya pattern with bilateral occlusive disease of the terminal segments of the internal carotid arteries and associated basal collaterals. Perfusion studies marked hypo-perfusion of the left hemisphere. | Corticoids, Azathioprine, MMF, CYC, frozen plasma | c.287del | Gly96Alafs / Complete | [19] |  |
| 1/M/9 | Iraq | Malar rash, LN (Type II), fever | CNS involvement, non-specified (Lethargy, difficulty to walk) | Recurrent infections  Dead at 9, 4 months after allo-HSCT | ANA +  SSA +  RNP + | ND | MRI: Contrast enhancement in the left putamen in T1-weighted sequences. Enhanced signal in the right basal ganglia and capsula interna. | Rituximab, frozen plasma, plasma exchange  Allo-HSCT | ND | Gln208X / Complete | [5] |  |
| 1/M/24 | Dutch | Malar rash, photosensitivity, oral ulcers, arthritis, LN (Type V) | Cognitive dysfunction, CVD | Recurrent infections | ANA +  DNA -  SSA+  Sm +  RNP + | C1q=low 20%  CH_50_=0  AP_50_=N  C3/C4=N  MBL=N  C3c and C5b9=N | MRI: Multifocal diffuse grey matter hyperintensities located in the fronto-temporal right lobe and high-intensity area on T2 in multiple regions of the right frontal and parietal lobes with high-intensities on the diffusion weighted imaging study. CT-angiography: no signs of cerebral vasculitis. | Corticoids, CYC | g.5499G>A | Gly34Arg / Dysfunctional | Present case |  |
| ACS: acute confusional state; CYC: cyclophosphamide; IVIG: intravenous immunoglobulin therapy; LN: lupus nephritis; MMF: mycophenolate mofetil; ND: non described; NPSLE: neuropsychiatric systemic lupus erythematosus | | | | | | | | | | | |  |
